# Applied Practice and Possible Leverage Points for Information Technology Support for Patient Screening in Clinical Trials: Qualitative Study

**DOI:** 10.2196/15749

**Published:** 2020-06-16

**Authors:** Linda Becker, Thomas Ganslandt, Hans-Ulrich Prokosch, Axel Newe

**Affiliations:** 1 Chair of Health Psychology Friedrich-Alexander University Erlangen-Nürnberg Erlangen Germany; 2 Department of Biomedical Informatics Heinrich-Lanz-Zentrum Mannheim Germany; 3 University Medicine Ruprecht-Karls University Heidelberg Heidelberg Germany; 4 Chair of Medical Informatics Friedrich-Alexander University Erlangen-Nürnberg Erlangen Germany

**Keywords:** clinical trial, patient screening, electronic support, clinical information systems, inclusion criteria, exclusion criteria, feasibility studies, mobile phone

## Abstract

**Background:**

Clinical trials are one of the most challenging and meaningful designs in medical research. One essential step before starting a clinical trial is screening, that is, to identify patients who fulfill the inclusion criteria and do not fulfill the exclusion criteria. The screening step for clinical trials might be supported by modern information technology (IT).

**Objective:**

This explorative study aimed (1) to obtain insights into which tools for feasibility estimations and patient screening are actually used in clinical routine and (2) to determine which method and type of IT support could benefit clinical staff.

**Methods:**

Semistandardized interviews were conducted in 5 wards (cardiology, gynecology, gastroenterology, nephrology, and palliative care) in a German university hospital. Of the 5 interviewees, 4 were directly involved in patient screening. Three of them were clinicians, 1 was a study nurse, and 1 was a research assistant.

**Results:**

The existing state of study feasibility estimation and the screening procedure were dominated by human communication and estimations from memory, although there were many possibilities for IT support. Success mostly depended on the experience and personal motivation of the clinical staff. Electronic support has been used but with little importance so far. Searches in ward-specific patient registers (databases) and searches in clinical information systems were reported. Furthermore, free-text searches in medical reports were mentioned. For potential future applications, a preference for either proactive or passive systems was not expressed. Most of the interviewees saw the potential for the improvement of the actual systems, but they were also largely satisfied with the outcomes of the current approach. Most of the interviewees were interested in learning more about the various ways in which IT could support and relieve them in their clinical routine.

**Conclusions:**

Overall, IT support currently plays a minor role in the screening step for clinical trials. The lack of IT usage and the estimations made from memory reported by all the participants might constrain cognitive resources, which might distract from clinical routine. We conclude that electronic support for the screening step for clinical trials is still a challenge and that education of the staff about the possibilities for electronic support in clinical trials is necessary.

## Introduction

### Background

Clinical trials are one of the most challenging and most meaningful designs in medical research. Many instructions on how to design a clinical trial optimally can be found in the literature [1,2]. However, the most challenging and essential steps before a clinical trial can start are the phases of feasibility estimations and patient prescreening. The latter includes the identification of patients who fit the study design and who fulfill the inclusion criteria but do not fulfill the exclusion criteria. The screening step (ie, both feasibility screening and patient prescreening) for clinical trials might be supported by modern information technology (IT). Hospitals and other research organizations are challenged with regard to establishing appropriate IT architecture [3].

### Challenges in Patient Screening and Recruitment

It is well known that patient recruitment is a crucial factor for the success of a clinical trial and that failing to achieve recruitment objectives is a common problem [4,5]. This was first reported in 1984 [6], but it has not changed significantly until very recently [7]. For example, McDonald et al [8] found that only 31% of systematically reviewed trials recruited to 100% of their original target and that 45% failed to recruit to within 80% of the target.

A lot of research has been conducted on unveiling and discussing typical problems with regard to achieving planned recruitment goals [9]. Several strategies to improve recruitment have been analyzed [8,10-12], but the specific solutions from individual trials are not easily generalizable [5]. Some major problems are the work overload of staff and their lack of time with regard to patient recruitment [4,13]. Therefore, if electronic support (ie, support by IT, see section Prior Work on the Analysis of Workflows for Screening and Recruitment) accelerated the recruitment procedure, it could relieve the staff of some of these duties. However, a common problem is that the staff have no access to and no awareness of relevant trial information, which is often available via paper-based documents only [4,14]. A further problem is that clearly defined, unambiguous inclusion and exclusion criteria for eligibility are often missing [4]. Furthermore, inclusion and exclusion criteria are often described in free text, making the mapping to electronically available data difficult [4,14].

### Prior Work on the Analysis of Workflows for Screening and Recruitment

Although many papers regarding the support of clinical trials by means of IT have been published, most of them present prototypes or stand-alone solutions [15-17]. Many authors agree that the impact on the routine workflow of the involved staff needs to be kept as low as possible and that understanding these workflows is crucial [4,18,19]. However, little research has been conducted on these routine workflows outside the setting of site-specific solutions. Furthermore, only a few studies dwell on the involved actors or roles. A precise description of a routine workflow was published by Embi et al [20,21], and it included the involved physicians, main investigators, and clinical research assistants. Another analysis of the trial management workflow of oncological phase III and phase IV trials at 2 sites in São Paulo and Rio de Janeiro was conducted by de Carvalho et al [22]. They found a lack of standardized processes for data capture, a multiplicity of data repositories, and a shortage of decision support systems. They concluded that workflows need to be reorganized to use IT more efficiently and that standard procedures need to be established. Trinczek et al [23] modeled workflows in a more formal way using the Business Process Model and Notation based on unstructured interviews and concluded that complexity could lead to redundant work by an investigator and a study nurse performing the same steps twice with the same patient.

Moreover, a strong focus on both the role of physicians and on the medical field of oncology can be observed in previous research [24]. Although the role of physicians is very important [20], they are not the only party involved in the bedside care of patients. Nurses and dedicated trial personnel also need to be considered.

### Motivation and Aims

There are well known and still-existing problems with regard to screening and recruiting patients for clinical trials. Clinical information systems (CIS), electronic health records, and, more generally, hospital information systems (HIS) are often considered as suitable tools for supporting the process of trial management [4,9,18]. Any IT-based solution must fit into the respective workflows [4,18,19], but these workflows—and in particular, the routine of the bedside staff that use these IT systems—have largely remained without investigation so far.

This qualitative and explorative study aimed (1) to evaluate which tools for feasibility estimations and patient screening are actually used in clinical routine and (2) to evaluate which method and type of IT could support the clinical staff. The findings are intended to lay the foundation for a larger, more representative, and more structured study.

We focused on the real-world implementation of workflows, regardless of theoretical or predefined models, as described in the study by Lee et al [14]. To achieve these goals, we conducted semistandardized interviews in a German university hospital.

Keeping in mind that trial management is a complex process and likely to require individual approaches fitted to the respective setting, we aimed to include a variety of medical disciplines and a variety of medical staff. Therefore, we considered personnel who were directly involved in screening and recruiting patients for clinical trials, with a strong focus on the bedside staff. We assumed that the bedside staff are usually burdened with nontrial-related duties and, therefore, are most likely hampered by suboptimal workflows.

## Methods

The report of this study is based on the Consolidated Criteria for Reporting Qualitative Research [25].

### Recruitment of Interviewees

To achieve the best possible variation (in age, gender, role, experience, and medical discipline), all clinics of a German university hospital were contacted by email, the project was briefly outlined, and participation was kindly requested. Of the 25 clinics, 11 responded, and 5 out of these 11 declined (either because of studies not being undertaken at the facility or without further explanation). Appointments were arranged with 5 of the remaining 6 clinics; 1 clinic canceled later.

Interviews were conducted with 5 participants (1 per clinic, 2 males, mean age 37.2, SD 7.9 years), each from a different ward (cardiology, gynecology, gastroenterology, nephrology, and palliative care). One of the interviewees was personally known to author AN on a professional level from a previous collaboration. Out of the 5 participants, 4 were directly involved in patient screening. Three of the interviewees were physicians, 1 was a research assistant, and 1 was a study nurse. The variety of interviewees was deemed sufficient for a qualitative study; therefore, no further efforts were made to increase the number.

Written and informed consent was obtained from all participants. The local ethics committee of Friedrich-Alexander University Erlangen-Nürnberg approved the study.

### Data Collection

Semistandardized interviews that comprised 4 parts were conducted in a one-to-one setting (interviewer-interviewee). The location was chosen by the interviewees; in all cases, it was an undisturbed office environment. All participants had taken sufficient time and were not under time pressure. Each interview lasted approximately 30 min and was conducted in German by author AN, who was a PhD student in medical informatics at the time of the interviews. All interviewees were native German speakers. Before the interviews, the participants were informed about the purpose of the interview and the research context.

The interviews were voice recorded electronically using the built-in recording app of a smartphone and were then transcribed and anonymized before analysis. Written notes were not taken, but a short questionnaire with demographic variables was filled out by the interviewees. The interviews were divided into the following 4 parts: (1) the actual state of study feasibility assessment, (2) the actual patient prescreening strategy, (3) the actual IT support for feasibility estimation and prescreening, and (4) the request for IT support. The interview guidelines can be found in Multimedia Appendix 1. According to the qualitative approach of this study, no a priori hypotheses were developed or considered subsequently.

### Data Analysis

The interview data were analyzed by 2 independent raters (authors AN and LB)—native German speakers— who were both postdoctoral researchers (author AN, male: medical informatics and author LB, female: health psychology) at the time of the analysis.

The analysis was based on the original German transcripts. It was carried out as follows. First, both raters independently identified and coded statements (quotes) in the transcripts that belonged to one of the research questions. Second, the coded statements were compared, and matches among the interviewers were collected in a separate document. Mismatches were discussed until consensus was reached. Next, based on discussions between the authors, clusters of similar statements were determined, and general terms for the categories were agreed upon. Subsequently, the frequencies of each category were determined. These categories as well as exemplary statements are provided in the Results section.

Finally, German quotes were translated into English for publication. (Note: Naturally spoken language is difficult to translate, especially if it comes from free speech. Therefore, the translated quotes may be *bad English*. However, they were already *bad German* in the first place, and the authors intended to keep the authenticity of the quotes, which naturally goes hand in hand with linguistic errors.) The data analysis, however, was based on the original German texts and was performed by native German speakers only.

## Results

The original interviews were transcribed to 27,985 words, from which 193 key statements (quotes) were extracted. All citations and classifications are provided in Multimedia Appendix 2.

### User Statistics

Out of the 5 interviewees, 4 were directly involved in patient screening. The other interviewee was a clinician who was responsible for study co-ordination and who delegated the screening to a study nurse. The percentage of work time that was spent screening for study participants ranged between 1% and 100% (mean 28.7%, SD 40.4%). The number of actual clinical trials ranged between 0 for the research assistant and 25 for 1 clinician (mean 12.8, SD 11.0). The number of inclusion and exclusion criteria per study ranged between 10 and 28 for each. The percentage of standard inclusion and exclusion criteria that remained the same for each study was estimated to be approximately 70% (range 50%-100%). The average number of patients who had to be recruited within 1 month was estimated to be 30.8 (SD 31.0; minimum 5, maximum 73, and 1 missing). A high-level coordination office was present in 3 of the 5 wards.

### First Interview Part: Study Feasibility—Current Situation

In the first part of the interviews, the participants reported how they get to study feasibility estimations, that is, how they assess if there are enough patients available who fulfill the inclusion criteria but do not fulfill the exclusion criteria. An overview of the categorized answers and their frequencies is provided in Table 1.

**Table 1:** Answer categories in part 1 of the interviews in which the interviewees reported how they get to study feasibility estimations (N=5).

Category number	Category	Value, n^a^ (%)
1.1	Experience	3 (60)
1.2	Internal statistics	3 (60)
1.3	Gut feeling	3 (60)
1.4	Works (very) well	3 (60)
1.5	Estimations	2 (40)
1.6	From memory, in mind	2 (40)
1.7	Ask colleagues or other wards	2 (40)
1.8	Literature search	2 (40)
1.9	Automatically from memory	1 (20)
1.10	Searching in protocols	1 (20)
1.11	Extrapolation from previous years’ data (problem: bad documentation so far)	1 (20)
1.12	Parallel to the clinical routine	1 (20)
1.13	Personal exchange between clinicians, coordinators, central coordinators, and bedside staff	1 (20)
1.14	Search in databases if the study is important	1 (20)
1.15	Very time consuming	1 (20)
1.16	We have no search engine	1 (20)
1.17	Looking in existing pool of patients	1 (20)
1.18	Difficulties	1 (20)
1.19	Underestimations	1 (20)
1.20	Sometimes overestimations and sometimes underestimations	1 (20)
1.21	Sometimes good, sometimes bad, or sometimes average	1 (20)
1.22	Need for exact estimates	1 (20)
1.23	Problems when not documented	1 (20)
1.24	No quality management	1 (20)
1.25	Error prone	1 (20)
1.26	Pessimistic guessing	1 (20)
1.27	Create a documentation of included and excluded patients	1 (20)

^a^The frequencies indicate the number of interviewees out of 5 who gave answers that fit into the category.

All interviewees reported that most of the feasibility estimation is done from memory or is a *gut feeling* and that it is mostly based on experience:

Everyone goes through the complete patient lists in his mind, you sometimes have redundancies, but nevertheless you get a very good result.

Quote #1

Furthermore, it was reported that a comparison with previous studies is performed from memory or in databases:

I just look at the numbers of [year] and do my queries, using Access.

Quote #24

This offers good results for studies with similar inclusion and exclusion criteria, but it works rather poorly for new types of investigations with different criteria. Two of the interviewees rated the actual procedure as good.

Only 1 interviewee reported an ongoing documentation of feasibility estimations in a separate file:

Then I would create a separate documentation in research, where I document all the patients that we have on the ward and I just for each patient then note to what extent the inclusion and exclusion criteria applied that I have come to the conclusion that we are trying to integrate or not so that later.

Quote #35

Furthermore, the study nurse reported that screening lists are created in which all screened patients and the reasons for inclusion or exclusion are listed. One interviewee reported that the current procedure tends to result in underestimations. Another interviewee stated that it depends on the study of how good the procedure works and if it results in underestimations or overestimations. The other interviewees did not comment on this.

Most of the participants reported an active exchange among study nurses and clinicians from the same ward and from other wards for assessing feasibility estimations irrespective of the responsibilities (ie, the study nurses reported that they ask the clinicians and vice versa). Moreover, 1 interviewee reported extrapolation from previous years’ data based on specific databases. However, he also mentioned that some therapy situations have not been well documented, which makes the feasibility estimations difficult.

Furthermore, 1 interviewee reported doing an extensive literature search for getting prevalence rates and extrapolating this for the patient numbers in the actual department:

Then we extrapolate the current year.

Quote #11

Overall, the actual state of study feasibility estimation was dominated by human communication and estimations from memory. Success mostly depended on experience. Electronic support had only little importance so far.

### Second Interview Part: Patient Screening—Current Situation

In the second part of the interviews, participants were asked how eligible patients for recruitment were identified for active clinical trials. An overview of the answer categories and the frequencies is provided in Table 2.

Again, most of the participants reported that most of the screening procedure is done from memory:

Because it all happens in mind.

Quote #49

And that it is best to memorize every study as well as all inclusion and exclusion criteria:

It is best if I know all the studies that are currently running in our ward.

Quote #77

Moreover, 1 interviewee stated:

In principle, it all depends on me, both the selection, the thinking about which patient exists, which patient might be suitable, contacting patients, including patients yes or no, and continuing to look after the patients, all my job.

Quote #53

Three interviewees felt that screening of patients requires much effort, as expressed by 1 of them:

That is an additional effort, exactly, which is usually not paid for. [...] So, it is usually the case that these studies run alongside the normal work of the doctors. They are all working to capacity anyway.

Quote #61

One interviewee stated that for restrictive inclusion criteria, success is “a matter of luck” [Quote #74].

Furthermore, 2 of the interviewees named announcements in local newspapers and postings in local offices as a strategy for recruiting. This procedure is chosen, especially, if an external sponsor is involved. However, the success rate is rather low because in most cases, only a tiny fraction of the participants actually responds to newspaper announcements. However, 2 of the interviewees reported that some of the patients come on their own initiative.

Three interviewees reported that the most important and successful strategy for recruitment is (1) asking the inpatients or outpatients during regular ward rounds or (2) through personal conversation with colleagues (Quote #96: “...quite simply the personal conversation on the ward.”).

In contrast, 1 interviewee reported no division of labor and that there is 1 dedicated employee per study, who is responsible for the entire workflow. He also reported that an alert from clinical personnel does not work well:

You have to take care of yourself every day, you get no patients reported [...].

Quote #100

Regarding IT usage, 1 interviewee stated:

Earlier it worked, and recruiting many years ago was [...] significantly better without all the systems.

Quote #93

Two of the interviewees reported that they actively search in the CIS. One reported searching in paper-based patient records or in Microsoft Word documents (when data are not available in the CIS and because free-text searches are not possible in the CIS) and that the applied strategy and effort depend on the study:

We have a wide variety of examinations, and then every single examination really matters, what kind of people do I actually need? The search for patients is correspondingly time-consuming.

Quote #55

One clinician reported that he uses a *study book* for some studies, which is kept up to- date by the clinicians and to which the study nurses have access to:

This is a study book, where the current studies from each year are always included. It is reissued once a year. There are inclusion and exclusion criteria.

Quote #82

Three interviewees mentioned paper-based reminder notes with the inclusion and exclusion criteria, which can be found in the treatment rooms. Another screening strategy mentioned by the study nurse was that the clinician asks the team if there is an eligible study for a specific patient. Furthermore, it was reported that regular team meetings and regional meetings take place in which inclusion and exclusion criteria are discussed and where it is decided which patients are potentially eligible for inclusion:

But especially for the prescreening nothing is documented, that is, everyone does it for himself [...] and thinks about how many patients should be included, who that would be, and this will then be gathered in the team meeting. During brainstorming, everybody thinks about who should be included and at a team meeting, which we have relatively often, which we always have regularly, all patients that could be included are put forward.

Quote #50

**Table 2:** Answer categories in part 2 of the interviews in which the interviewees were asked about the actual state of their screening strategy (N=5).

Category number	Category	Value, n^a^ (%)
2.1	This is done in mind	4 (80)
2.2	Personal contact, actively asking ambulatory and inpatients	3 (60)
2.3	The clinician asks the team whether there is an eligible study for a specific patient	3 (60)
2.4	Personal motivation is the most important factor for successful screening	3 (60)
2.5	Printed IC^b^ and EC^c^ as reminder notes, handouts	3 (60)
2.6	Works well	3 (60)
2.7	Much effort	3 (60)
2.8	Regular team meetings	2 (40)
2.9	Patients come on their own	2 (40)
2.10	Active search in CIS^d^	2 (40)
2.11	Works not well	2 (40)
2.12	Announcements in local newspapers	2 (40)
2.13	Postings and flyers in local offices	1 (20)
2.14	Internally filtering of the inpatients (in mind)	1 (20)
2.15	It all depends on me	1 (20)
2.16	Search in paper-based records	1 (20)
2.17	Written documentation of patient screening strategy and reason for inclusion or exclusion	1 (20)
2.18	Initiated by sponsors	1 (20)
2.19	Not using the CIS	1 (20)
2.20	Announcements in specialist journals	1 (20)
2.21	By the sponsors themselves	1 (20)
2.22	Preselection by the study nurses	1 (20)
2.23	Not much effort	1 (20)
2.24	No regular team meetings	1 (20)
2.25	Error prone, cannot have all in mind	1 (20)
2.26	Depends on the study	1 (20)
2.27	50% in mind	1 (20)
2.28	Excel sheets with contact information	1 (20)
2.29	Matter of luck	1 (20)
2.30	Cooperation with residents	1 (20)
2.31	Scheduling program	1 (20)
2.32	Study book	1 (20)
2.33	Printing out the study book entries	1 (20)
2.34	Back then, it worked better (without IT^e^)	1 (20)
2.35	Previously known patients	1 (20)
2.36	I am solely responsible	1 (20)

^a^The frequencies indicate the number of interviewees out of 5 who gave answers that fit into the category.

^b^IC: inclusion criteria.

^c^EC: exclusion criteria.

^d^CIS: clinical information systems.

^e^IT: information technology.

Only 1 interviewee reported a written documentation of the patient screening strategy, including the reason for inclusion or exclusion. Others stated that screening protocols would definitively be helpful, but they are not feasible because of time pressure. One interviewee stated that the burden for patient screening depends on the study: inpatients with high care expenses are good to be screened, but screening in clinical routine is often forgotten.

Most of the interviewees stated that the most relevant factor for successful screening is personal motivation and not the specific tools that are used for this reason:

Extremely important [...] it takes a lot of heart and soul.

Quote #77

To summarize, the actual state of the screening procedure was dominated by human communication and estimations from memory. Electronic support was used but with little importance so far. Overall, most of the interviewees saw the potential for improvement, but they were also largely satisfied with the outcome of the current approach:

I don’t think it’s very modern to do it that way.

Quote #98

I think that patient identification works well for us. I’m not even dissatisfied with it. I believe that this is also a cumbersome way and very time consuming, but ultimately the result fits the outcome.

Quote #104

### Third Interview Part: Information Technology Support—Current Situation

In the third part of the interviews, interviewees were asked which kind of IT support they use in clinical routine and, especially, for patient screening. An overview of the answer categories and frequencies is provided in Table 3. All participants reported some kind of IT support. Four interviewees reported that they regularly search in the CIS. However, 1 interviewee stated that he does not use electronic systems in most cases:

No, not in clinical information systems.

Quote #120

...we’re not searching in [CIS]. So, I don’t think that has ever been done in our house...I’ve never tried that myself, and I think in our ward...nobody does that.

Quote #122

However, he sometimes searches in Excel (Microsoft) lists (databases) for specific diagnoses:

Then there is a small database for each clinical picture; partially it’s just some Excel lists or something.

Quote #121

Furthermore, he used an electronic data capture system for 1 study, but this is not permitted for other studies because of data protection policies:

We only used it relatively rarely, and there are political reasons for that. [...] Because, we have assured the patient that we will not pass on their data so that we cannot simply give it to anyone [...]. So, we explicitly promised the patient that we would not do this.

Quote #127

Two more interviewees reported having ward-specific patient registers for specific diagnoses and with comprehensive entries (eg, blood samples) as well:

We have a [specific diagnosis] register, there the patients are recorded relatively comprehensively, with blood samples and everything. And then you can also research it. We all have it in there.

Quote #105

I just print out the whole [regular meeting] up here on a sheet of paper and go through all the patients. Most of the time I select over 60 patients.

Quote #87

These have been specially built for clinical trial screening by in-house research groups. The main reason why 1 interviewee does not use electronic databases regularly is that the inclusion and exclusion criteria are very complex and:

Could be operationalized [in principle], but [that] would be an insane effort.

Quote #110

The main problem that he has with the CIS is that the diagnoses do not necessarily match the diagnoses in the paper-based records:

I have of course already selected according to diagnoses in [CIS]. However, these diagnoses are not necessarily the diagnoses that I now have in a doctor’s letter. That is the problem.

Quote #115

However, he stated that:

The good thing is...what we are always interested in...for example that the whole blood values can be seen at a glance. This is important for us if we go through such inclusion and exclusion criteria.

Quote #119

However, he also stated that:

In the end it is always the case that we have to read the doctor’s letter again.

Quote #117

One interviewee stated that solid numbers (eg, from blood samples) make little sense in his case because operational reports are of greater importance. He also noted that he searches only sparsely in the CIS. He mentioned that there is a lot of data in free-text medical reports, but screening the whole text is too time consuming and that the doctor’s letters “...cannot be evaluated at all” (Quote #111). Another problem that was mentioned is that a specific diagnosis is not documented in many cases. Moreover, it is not documented if the patient already participates in another study.

**Table 3:** Answer categories in part 3 of the interviews in which the interviewees reported about the actual state of information technologies support for patient screening and feasibility estimations (N=5).

Category number	Category	Value, n^a^ (%)
3.1	Active search in a CIS^b^	4 (80)
3.2	Search in electronic records (Word documents), directory with findings from the examination	3 (60)
3.3	Search in databases (eg, Excel files)	3 (60)
3.4	Ward-specific patient register (very extensive)	3 (60)
3.5	Much effort	2 (40)
3.6	Not enough or not much data are collected electronically	2 (40)
3.7	Electronic patient lists	1 (20)
3.8	Beneficial	1 (20)
3.9	We do not search in the CIS	1 (20)
3.10	Do not know the CIS	1 (20)
3.11	Complex data not in the database	1 (20)
3.12	Do not have a database	1 (20)
3.13	Problem: diagnosis in the doctor’s letters does not match the entry in the CIS	1 (20)
3.14	At the end, using the (paper-based) doctor’s letters	1 (20)
3.15	Ward-specific solution	1 (20)
3.16	Concerns with data protection policies	1 (20)
3.17	Electronic scheduling program	1 (20)
3.18	In the CIS, certain information is taken over from the last entry	1 (20)
3.19	Database with recruiting numbers	1 (20)
3.20	Feasibility estimations in internal database	1 (20)
3.21	Problem: do not have access to the CIS	1 (20)
3.22	Laboratory-specific database	1 (20)
3.23	No electronical doctor’s letters	1 (20)
3.24	Milestone	1 (20)
3.25	Difficult at the beginning	1 (20)
3.26	Works well	1 (20)

^a^The frequencies indicate the number of interviewees out of 5 who gave answers that fit into the category.

^b^CIS: clinical information systems.

Furthermore, it was reported by 1 interviewee that at his ward they:

Produce recruitment numbers from our studies once a month, where we have our clinical database where all study patients are registered.

Quote #134

One problem that was raised by 1 of the interviewees was that he had difficulties getting access to the CIS and other databases:

We [...] have difficulty accessing this electronic data.

Quote #138

Often we don’t get any rights to see this, so even if we ask that we only have read rights—we don’t want to document anything in the patient record, that’s totally okay, but we would like to be able to read it.

Quote #139

To summarize, there are already a few, mostly self-developed, solutions, but most of them are only partially used or have the potential to be improved:

Everything has grown historically, and these working groups have been on the road for many years and they almost always work with their own databases.

Quote #125

### Fourth Interview Part: Information Technology Support—Request From Staff

In the last part of the interviews, the interviewees had the opportunity to express their requests for future IT support. An overview of the interview answers is provided in Table 4. The initial, spontaneous answers of 3 of the 5 interviewees were that it is not easy or not realistic to use or to develop an IT tool that is really helpful:

Not easy...Not easy...

Quote #146

I think that the things that you really need cannot be implemented at all, so they really cannot be implemented.

Quote #169

I don’t know of any tool that would make work easier, right?

Quote #175

However, after the interviewees were given time to think about the potential of IT support, the interest and need for electronic support became more apparent to them. After a period of consideration, most participants stated that it would be helpful to have an electronic database where one could search and enter criteria and which creates a list with patient proposals:

Definitely, because we have so many patients. And if there was such a thing or if I could wish for something: we take over certain criteria that are stored operationalized.

Quote #147

[...] Every patient who has a main diagnosis like this, of course, has to appear somewhere in a database field and with a YES / NO query or whatever, that you can select that. But there are possibilities that would help us extremely.

Quote #154

For some things, I don’t find it wrong to search in the hospital information system.

Quote #171

So, there would be many options. A clever mind would have to sit behind it and go through it individually with the colleagues from the [department] and then quasi operationalize it. And then a database. Then you could do a lightning search.

Quote #156

This would be appreciated for both study feasibility estimation as well as for patient prescreening.

Regarding whether self-paced or proactive approaches would be preferred, the interviewees stated that both approaches have advantages and that this depends on the study and the number of eligible patients. An interviewee said of a self-paced approach:

If of course you had a huge file now if I had a huge file where I could enter certain things and it would at the end vomit the patients who met all of these criteria, that would of course be fantastic.

Quote #163

Another interviewee said of a proactive approach:

It would of course be really convenient if I didn’t have to search through this electronic documentation myself anymore, but if there was a programmed tool that simply queries certain fields and data at defined times or in defined periods, and whenever there is just a certain result comes out, I get a “pling” on the screen, [...] That would be extremely great.

Quote #183

The main concerns were too many alerts for the proactive solutions, but with a flexible alert time that depends on the study, it would be an acceptable solution:

Because I think the problem of these 10-minute memories or something becomes relevant if you recruited at many stations, [...] It might just be nicer if you bundled it up and said you get 2 in the morning and at noon sometimes a report bundled with everyone, but for [...] critical patients it would be totally okay for [...].

Quote #192

However, for patients who are time constrained it would be absolutely acceptable to get several alerts a day:

But if that were continuously and I was notified at all times, that would be a major advantage.

Quote #189

That would be very profitable, precisely because we expect fast progress and rapid changes in the general condition.

Quote #190

However, it was also stated that an IT system would be too time consuming and would lead to additional work:

Very few things will run so automatically that you make a request and that the system is completely there in its perfection, i.e. it takes a lot of time and money.

Quote #175

For example, it would be very important to keep the data (eg, the main diagnosis) up to date, but that does not always happen or takes too long:

The main diagnosis must always be kept up-to-date, and that would of course be important to keep the doctor’s letters up-to-date.

Quote #152

Furthermore, all data would have to become operationalized, which is also not always possible:

*And these are recurring inclusion/exclusion criteria in many studies, which are very similar, of which itcould be operationalized, but would be an insane effort.* [Quote #110]

[...] There is always a certain limitation of course there is always.

Quote #168

A further concern was to get interdepartmental data access and not only ward-specific access:

[...] There would be a significant added value if we could ensure that there were cross-departmental collaborations.

Quote #187

Moreover, 1 interviewee complained that he does not have access to patient data from local offices with which they co-operate, and this is because of data protection policies:

So especially if you work together with private practices, we cannot access their data, because their computers with patient data are not connected to the network for data protection reasons.

Quote #170

**Table 4:** Answer categories in part 4 of the interviews in which the interviewees were asked for their request for information technologies support (N=5).

Category number	Category	Value, n^a^ (%)
4.1	Database (tool in which some criteria [eg, main diagnosis] could be entered and which creates a list with patient proposals)	4 (80)
4.2	Proactive system	4 (80)
4.3	Passive system	4 (80)
4.4	Not easy, not realistic	3 (60)
4.5	Would be helpful/beneficial/fantastic	3 (60)
4.6	Interesting project, would like to learn more	2 (40)
4.7	I have concerns with regards data protection	2 (40)
4.8	Additional work, much time effort	2 (40)
4.9	Problem: difference between easy and complex cases/studies (number of IC^b^ and EC^c^), need for specific solutions	2 (40)
4.10	Active or passive depends on the study and the number of available patients	1 (20)
4.11	Would be time saving	1 (20)
4.12	Access to data from local offices	1 (20)
4.13	Databases have to be kept up-to-date, and this is not always possible in clinical routine	1 (20)
4.14	Merging (in house) interfaces	1 (20)
4.15	(Eventually) too many alerts for the proactive	1 (20)
4.16	For time-critical patients, it would be absolutely okay to get several alerts a day	1 (20)
4.17	Voice recognition software for the creation of doctor’s letters	1 (20)
4.18	Certain limitation	1 (20)
4.19	Do not know a tool that could facilitate work	1 (20)
4.20	Do not really need it	1 (20)
4.21	It takes a lot of time and money	1 (20)

^a^The frequencies indicate the number of interviewees out of 5 who gave answers that fit into the category.

^b^IC: inclusion criteria.

^c^EC: exclusion criteria.

An interesting idea proposed by 1 of the clinicians was that it would be extremely helpful to have a voice recognition software for the creation of doctoral letters.

Overall, most of the interviewees showed interest in the topic and said that they would like to learn more about the possibilities of how IT could support and relieve them in their clinical routine:

...which would of course make it easier if we could get to know these systems.

Quote #181

So, it only benefits everyone. Win-win situation, there is nothing that would be a disadvantage.

Quote #157

I think it’s a very interesting project.

Quote #158

## Discussion

### Summary of Principal Findings

This study aimed to obtain insights into which tools for feasibility estimations and for patient screening are actually used in clinical routine by means of a qualitative approach. Furthermore, we were interested in finding possible leverage points for using IT to support clinical staff. Overall, the actual state of study feasibility estimation and the screening procedure were dominated by human communication and estimations from memory. Electronic support was used but with little importance so far. Searches in ward-specific patient registers (databases) and searches in CIS were reported. Furthermore, free-text searches in medical reports were mentioned. Most of the interviewees saw the potential for improvement in the actual systems and were interested in learning more about the possibilities of how IT could support and relieve them in their clinical routine.

### Electronic Support for Study Feasibility

All interviewees reported that most of the feasibility estimations are done from memory and that currently IT support plays only a limited role. This is surprising because if an up-to-date database of all patients from previous years with the most relevant inclusion and exclusion criteria was available, a simple, time-saving search could give good estimations [26,27].

One problem that was often raised was that in many cases, not all exclusion and inclusion criteria are known, making it challenging to receive acceptable estimations independent of the availability of IT support. On the other hand, it was mentioned that there are sometimes too many exclusion and inclusion criteria that cannot be remembered accurately. This is an easy-to-implement aspect where electronic support could begin, for example, with a defined set of data elements [28]. Most of the interviewees reported that feasibility estimations have not been well documented so far. If an electronic search was performed, this strategy could be saved, making it possible to verify these steps later when needed (eg, for publications). Therefore, we conclude that IT support for feasibility estimations appears to be beneficial to the clinical staff, but education about the possibilities of IT support is necessary.

### Electronic Support for Patient Screening

The interviewees reported that IT support currently plays a limited role in patient screening. Again, they reported that most steps are done from memory. However, all interviewees agreed that more data should be electronically available, and IT support would be very helpful if there was an up-to-date database. In this context, it should be mentioned that, before our study, the university hospital had been involved in a national project on IT-supported study recruitment and that the interviewed staff was completely unaware of this.

However, concerns about the possibility of a simultaneous search for several inclusion and exclusion criteria were raised. There was a need to search for 10 to 20 inclusion and exclusion criteria per case and to obtain a list of patients who fulfill most (but not necessarily all) of them.

Therefore, it is again concluded that familiarizing the personnel with modern IT solutions seems to be necessary. For example, Bache et al [29] developed a domain-specific query language as an interface between clinicians and stored data to facilitate this task on a technical level. A properly designed software tool has proven to be an enabler for users to correctly create and execute simple feasibility queries with only a relatively small amount of training [30]. Furthermore, several cost-benefit assessments have confirmed the benefits of electronic recruitment strategies [31,32].

### Electronic Support Strategies

In general, there are three strategies for patient identification and patient recruitment for clinical trials by means of HIS [18]: (1) systems that retrospectively query existing data in an HIS (Clinical Trial Recruitment Support Systems), (2) systems that monitor the occurrence of a specific event in an HIS to create some kind of alert (Clinical Trial Alert Systems), and (3) systems that require an operator to enter appropriate data to trigger an eligibility assessment.

The interviewees in our study did not prefer any of these approaches in general and clearly stated that it depends on the study (eg, the number of inclusion and exclusion criteria and if these can be operationalized easily) and, more importantly, on the patients who should be involved. For time-critical studies and rare patient groups, a proactive alert system was clearly requested. For more common patient groups with slow changes in health status, either passive systems or summarized alerts once a day or week were requested. Therefore, as has been reported previously, one of the key challenges in the design and operation of an active system is to find an operational model that has the least possible influence on the usual workflow of the target audience [18]. In addition, the threshold must be balanced carefully between a sensitivity that produces a high number of eligible patients and a specificity that avoids alert fatigue [4,15,20,21]. Several approaches and solutions have been presented in recent years [18]. Alerts can be sent out using paging systems [15,16], mobile devices [17], or emails [33,34] or can be sent directly within the HIS or the Clinical Decision Support System [13,20,21] or with a combination of both [35,36].

### Personal Motivation as a Key Factor

Most of the interviewees agreed that the success of screening depends—beside the complexity of the study and the support by appropriate IT systems—on the clinical staff and on their personal motivation. Therefore, a future challenge for the development of IT support systems seems to be finding IT solutions that motivate the staff to invest greater effort in screening without hampering their everyday work.

### Limitations

Our study provides numerous new insights into the work processes of the study staff directly involved in patient screening. However, our data were collected from 1 specific German university hospital, with a low sample size of 5. Therefore, the risk of bias needs to be considered, and our findings cannot be generalized to other institutions. Therefore, the next steps should be to repeat this investigation with other hospitals and within larger samples and a more structured approach. As the interviews were very time consuming and might have affected the clinical routine, a web-based survey might be a better means for this. The results of our study provide the basis for developing such a survey.

The raw data were collected in German, and the key quotes were translated into English for this publication. Naturally spoken language is difficult to translate, especially if it comes from free speech that is prone to grammatical errors and mental leaps. Therefore, parts of the content or the intention of the translated texts may have been lost in the translation. Hence, the translated quotes from the interviews that are published in this paper should be used with caution.

### Outlook

The results of our study cannot be generalized, but at least the following research questions could be derived from the findings and should, therefore, be addressed by future studies in the context of patient screening and recruitment:

- How well are the staff informed about the opportunities that IT offers in their work environment? How can the staff be educated in this regard?
- Does the staff already take advantage of IT support? Is this support based on tailored solutions or standard systems?
- How does the personal motivation of the staff affect the outcome? Would IT support have an impact (negative or positive) on this motivation?
- To what extent are estimates made from memory and why?
- How does the complexity of a study (eg, number of inclusion and exclusion criteria) affect the staff’s ability to estimate patient numbers correctly? Is there a threshold above which IT support makes sense? Is there a threshold below which IT support does not make sense?
- How does the diversity of IT systems in their work environment affect the staff’s ability to perform tasks for patient screening and recruitment?

In this context, the excerpts from the interviews provided in Multimedia Appendix 2 as well as the distilled answer categories provided in Tables 1-4 can serve as the basis for a questionnaire design.

Another aspect that should be addressed in future research is an inversion of the initial premise that electronic support would make the daily routine *easier* because the opposite case has not yet been considered at all. Although some interviewees expressed their concern that electronic support might not be *feasible*, none of them mentioned that it could be *obstructive*. Nevertheless, as this aspect was not explicitly addressed, it should be investigated or at least considered in follow-up studies.

### Conclusions

Although it seems that IT is nearly ubiquitous, our study suggests that IT support still has limited use in the screening step for clinical trials. Our main finding was that the staff is underinformed about modern IT solutions for the support of patient screening. This lack of IT usage and the resulting work-from-memory strategy might constrain cognitive resources, which might distract from clinical routine. Therefore, we conclude that it is necessary to educate the staff about the possibilities of IT support for clinical trial screening and—in addition to conducting a large-scale, more structured study based on our findings as proposed earlier—one future research option in this direction is to develop training programs that can achieve this goal.

## Appendix

Multimedia Appendix 1 Interview guideline and structure.

Multimedia Appendix 2 Extracted and categorized quotes from the interviews.

## Abbreviations

CIS: clinical information system
HIS: hospital information system
IMI: Innovative Medicines Initiative
IT: information technology
