# Supplementary material for: Applied Practice and Possible Leverage Points for Information Technology Support for Patient Screening in Clinical Trials: Qualitative Study
Source: JMIR Med Inform. 2020 Jun 16;8(6):e15749. doi: 10.2196/15749 (PMC7327588; doi:10.2196/15749)
Supplement: Multimedia Appendix 1 [file medinform_v8i6e15749_app1.pdf]

Appendix 1: Interview Guideline

Current Workflow:

Part 1: Study feasibility (current procedure)

1) Please describe how you estimate the number of patients who may be recruited for a planned clinical trial. [*Bitte beschreiben Sie, wie Sie zur Abschätzung von Patientenzahlen gelangen, die möglicherweise für eine geplante klinische Studie rekrutiert werden können*.]
2) How well do you think is this estimation in general? Do you tend to overestimate or to underestimate the numbers? [*Wie gut gelingt Ihrer Meinung nach die Abschätzung von Rekrutierungszahlen? Kommt es eher zu Über- oder eher zu Unterschätzungen?*]
3) When, where and to what extent do you have to search data in your own Hospital Information System to estimate the feasibility? To what extent do you estimate from memory? [*Wann, wo und in welchem Umfang müssen Sie für die Feasibility in Bestandsdaten recherchieren? Wie viel wird „aus dem Kopf“ gemacht?]*

Part 2: Patient identification (current procedure)

4) Please describe how your department identifies patients for the participation in clinical trials. Does this usually happen in the same way? How often do you deviate from your routine procedure? [*Bitte beschreiben Sie, wie in Ihrer Abteilung Patienten für die Teilnahme an klinischen Studien identifiziert werden. Läuft das im Normalfall gleich ab? Wie oft kommt es zu Abweichungen vom Regelfall?*]
5) When, where and to what extent do you have to search data in your own Hospital Information System to identify patients? To what extent do you identify patients from memory? [*Wann, wo und in welchem Umfang müssen Sie für die Identifizierung in Bestandsdaten recherchieren? Wie viel wird „aus dem Kopf“ gemacht?*]
6) Do you have to carry out any documentation when patients are identified? In what is this to be? Is there a duplicate data collection? [*Muss im Rahmen der Identifizierung von Patienten eine Dokumentation gepflegt werden? In welcher Form geschieht dies ggf.? Gibt es doppelte Datenerfassung?*]
7) Is there a division of labor in patient identification? Are the responsibilities clearly defined? Which tasks do you take on yourself? [*Gibt es eine Arbeitsteilung bei der Identifizierung von Patienten? Sind klare Zuständigkeiten festgelegt? Welche Aufgaben übernehmen Sie selbst?*]
8) Where do the patients for your studies come from? (own department, outpatients, other departments, tumor board) [*Woher kommen die Patienten für Ihre Studien? (eigene Abteilung, Ambulanz, andere Abteilungen, Tumorboard)*]
9) Are there regular meetings at which your department discusses topics related to patient recruitment? [*Gibt es regelmäßige Termine, bei denen Ihre Abteilung sich über Themen der Studienrekrutierung austauscht?*]
10) Does the patient identification constitute a temporary impediment to routine care? [*Stellt die Identifizierung von Patienten eine zeitliche Behinderung der Routineversorgung dar?*]
11) How successful do you think you are – on average – when it comes to the identification of patients? What are the problems, if there are any? What do you perceive as particularly annoying, disturbing or impedimentary? [*Wie gut gelingt Ihrem Gefühl nach die Identifizierung von Patienten im Durchschnitt? Was sind ggf. Probleme? Was wird als besonders lästig, störend oder behindernd empfunden?*]
12) How important is the "personal motivation" of the staff? How do you rate the general motivation in your department? [*Wie wichtig ist der Faktor „persönliche Motivation“ des Personals? Wie schätzen sie die allgemeine Motivation in Ihrer Abteilung ein?*]
13) How good is your knowledge about available studies? How effortful is it to keep up to date? [*Wie gut ist Ihre Kenntnis über verfügbare Studien? Wie aufwändig ist es, sich darüber auf dem Laufenden zu halten?*]

Appendix 1: Interview Guideline

14) Is there a kind of set of standard criteria that recur in many studies? [*Gibt es eine Art Satz von Standard-Kriterien, die bei vielen Studien wiederkehren?*]
15) How high is the percentage of the total patient population that you see personally? [*Welchen Anteil am gesamten Patientenaufkommen sehen Sie persönlich?]*

Part 3: IT support for patient identification/recruitment (current procedure)

16) Is the data in your hospital information system suitable for assisting the identification of patients? Why are they possibly not suitable? (Dummy diagnoses, delayed data capture, medical reports / free text documentation) [*Sind die Daten, die in Ihrem Krankenhausinformationssystem vorhanden sind, geeignet, um die Identifizierung von Patienten zu unterstützen? Warum sind sie ggf. nicht geeignet? (Dummy-Diagnosen, verzögerte Erfassung, Arztbriefe/Freitext-Dokumentation)*]
17) Is there already some form of electronic support for the identification of patients, e.g., a proper research database? Is it used? What is good with it, what is bad? Are there any experiences with alert fatigue? [*Gibt es bereits eine Form der elektronischen Unterstützung für die Identifizierung von Patienten, z. B. eigene Forschungsdatenbank? Wird sie genutzt? Was ist gut daran, was schlecht? Gibt es Erfahrungen mit Alert Fatigue?*]

Request:

Part 4: Request for IT support

18) In which form and by what means would you like to be supported? Would it be better on request or proactively (e.g., by alerts, paging, emails, messages in the hospital information system)? [*In welcher Form und über welchen Weg würden Sie gerne unterstützt werden? Lieber auf Anfrage oder proaktiv (z. B. Alerts, Paging, Mails, Meldung im Krankenhausinformationssystem)?*]
19) Which information about available studies would you like to get? [*Welche Informationen über verfügbare Studien würden Sie gern erhalten?*]
