# Supplementary material for: Applied Practice and Possible Leverage Points for Information Technology Support for Patient Screening in Clinical Trials: Qualitative Study
Source: JMIR Med Inform. 2020 Jun 16;8(6):e15749. doi: 10.2196/15749 (PMC7327588; doi:10.2196/15749)
Supplement: Multimedia Appendix 2 [file medinform_v8i6e15749_app2.docx]

| **Quote number** | **Quote (translated)** | **Category number** |
| --- | --- | --- |
| **Part 1: Study feasibility** | | |
| **Interview 1:** | | |
| **1** | If everyone goes through the compete patient lists in his mind, you sometimes have redundancies, but nevertheless you get a very good result, roughly, from all patients that might be included. | 1.6 |
| **2** | Very well. In fact, because most patients are already known. | 1.4, 2.6 |
| **3** | Hardly anyone falls out that could potentially be included. | 1.4, 2.6 |
| **Interview 2:** | | |
| **4** | I ask the senior physicians: How many cases do we have per year? Then they say a number either on a gut level or from memory. It’s only about a magnitude. | 1.1, 1.7, 1.13 |
| **5** | There are senior physicians who make statistics, how many interventions they make and when and in which area, and if they manage these statistics, they know, of course, that they have these numbers from the past few years in their heads or somewhere. I personally do not have access to these numbers, so I really have to go to the senior physicians and ask. | 1.2 |
| **6** | [How good are these estimations?] Quite well, I believe. | 1.4 |
| **7** | We took the findings from last year and searched for a keyword. | 1.10 |
| **8** | It is very time consuming for us. | 1.15 |
| **Interview 3:** | | |
| **9** | For IITs we do case number planning ourselves or in cooperation with the statistics department. | 1.2 |
| **10** | We then make estimates from experience of how many patients we can contribute. | 1.2 |
| **11** | We can roughly estimate how big the pool is we can resort to. | 1.2 |
| **12** | There is no search engine "vomit patients for me". | 1.16 |
| **13** | We have a large pool of patients and we know the people, so we can pretty well estimate it. | 1.17 |
| **14** | We had the greatest difficulties with a large epidemiological study, where it was much more difficult because we worked in cooperation with private medical practices, and that was much more difficult than expected. | 1.18 |
| **15** | Overall, this is a quite reasonable tool, this gut feeling. | 1.3, 1.4 |
| **16** | [What is more likely, over-estimations or under-estimations?] Under-estimations. | 1.19 |
| **17** | A lot is done from memory. | 1.6 |
| **18** | There is everything from good to inadequate, really depending on the clinical picture, the inclusion criteria and the time capacity of the study team. Of course, if they are more or less free, it works much better than if they have to do it on the side in everyday clinical practice. | 1.12, 1.21 |
| **19** | But since it is a functional working group with a study team that takes special care of it, it usually works at least satisfactorily. | 1.21 |
| **Interview 4:** | | |
| **20** | We first assess whether the study is so relevant to us that we have to determine the numbers exactly, or whether we estimate the numbers. | 1.5 |
| **21** | the smaller the number of patients or the estimated number of patients in screening or for randomization, the more precisely we need them in advance in order to be able to assess whether the study will bring us anything | 1.22 |
| **22** | We use data, databases, which, for example, if we are now studying [subject], we use the [specific database]. | 1.14 |
| **23** | There are approximately [number] primary cases that are associated with initial diagnosis [spec. Diagnosis] appeared in penultimate year. Then we extrapolate the current year. | 1.11 |
| **24** | I just look at the numbers of [year] and do my queries, using Access [database] | 1.14 |
| **25** | It becomes more difficult in therapy situations that are not documented | 1.23 |
| **26** | A second source would still be [central facility]. [This] also documents the patients regularly and in combination with [central facility] and our own [facility] we can, as it were, determine exact numbers | 1.2 |
| **27** | I then estimate based on my wealth of experience or if I don't get any further, if the queries don't help, an estimate is perhaps not plausible enough, then with the departing senior physicians, so to speak, who already are a little longer in our business and who estimate the patient numbers pi times thumbs. | 1.1 |
| **28** | I think that some studies are overestimated and some are underestimated, depending on how good the queries were and who you asked. | 1.20 |
| **29** | We have no feedback mechanism, we have no QM | 1.28 |
| **30** | This is really a pi-thumb-story. So, I think that some studies are overestimated and some are underestimated, depending on how good the queries were and who you asked | 1.5, 1.20 |
| **Interview 5:** | | |
| **28** | see if I have records that we have about our clientele, e.g. patient data from the clinic aggregated a year ago | 1.2 |
| **29** | Just see how many patients are included and how many are excluded that I can find an estimate | 1.5 |
| **30** | Then, I usually just use literature. | 1.9 |
| **31** | It is actually that I calculate it on the basis of my own internal data | 1.2 |
| **32** | Just calculate over a patient pool of one year or so with us and then orienting myself to it. | 1.2 |
| **33** | if I now, e.g. I need patients who are cognitively efficient, then sometimes I guess because that's not well documented, and then I guess | 1.5 |
| **34** | That is always a bit of intuition and gut feeling | 1.3 |
| **35** | This is of course very uncertain. You can really mess up with it. | 1.25 |
| **36** | Actually, quite good now, because of course your own experience increases and because your study experience also increases […] Because our patient clientele always has the same central criteria | 1.1 |
| **37** | That's why I always estimate pessimistically, always, that's part of my strategy […] Experience from colleagues who confirm that my pessimistic strategy is doing me well, that it is more realistically | 1.26 |
| **38** | I said I need patients who had the criteria over the years and when you find these patients, I need the and the data from them. And that worked really well. | 1.4 |
| **39** | So, this is always a separate documentation that does not enter the clinic. So, suppose I would now study the paper file or the electronic file, then I would all ... then I would create a separate documentation in research, where I document all the patients that we have on the ward and I just for each patient then note to what extent the inclusion and exclusion criteria applied that I have come to the conclusion that we are trying to integrate or not so that later, when I publish, I can also make a statement about the patients we did not include. | 1.27 |
| **Part 2: Patient recruitment – actual state** | | |
| **Interview 1:** | | |
| **40** | If there are drug trials, clinical trials that are supported by any sponsor, then they advertise in specialist journals and medical newspapers, and we have public postings. […] Pre-screening by the sponsors themselves who then speak directly to patients, and the patients contact us, by e-mail, by fax or via their [specialists], or via the general practitioners […] initiated by the sponsor. | 2.9, 2.12, 2.13, 2.18, 2.20, 2.21 |
| **41** | Internally, we do this by filtering patients based on certain criteria. | 2.14 |
| **42** | In the outpatient clinic, patients are sensitized by the doctors who work there, i.e. there are always team meetings. | 2.8 |
| **43** | And then the patient population is thoroughly screened by all active doctors in the outpatient care sector. | 2.8 |
| **44** | [...] our own team of study nurses, who also take care of pre-screening and pre-selecting the patients and then making a note in the files when the patient arrives. | 2.22 |
| **45** | So in the end we have 3: recruiting via manufacturers, sponsors from outside with advertising ... | 2.12, 2.13, 2.21 |
| **46** | So in the end I have to make the decision: If the patient is a) suitable for a study at all, b) of course I have to compare to all the patients I have in mind, those in the outpatient clinic, there might be some who could benefit from such a study, e.g. those who are largely therapy-refractory, those where nothing helped, and we are launching a new study, e.g. a drug study that could be offered to them. | 2.1 |
| **47** | However, one could theoretically look through the entire patient population with doctor's letters. | 2.12 |
| **48** | But most of what you could actually do, either just go through every doctor's letter from each patient; this is of course not feasible from a time investment point of view. That is why it is important to have as many patients as possible in your memory and to screen the entire throughput in your head, otherwise it would not be manageable in terms of volume; there are too many patients. | 2.1 |
| **49** | In the context of this pre-screening, it does not have to be documented theoretically, because it all happens in mind. | 2.1 |
| **50** | But especially for the pre-screening nothing is documented, i.e. everyone does it for himself, even from the doctors, and thinks about how many patients should be included, who that would be, and this will then be gathered in the team meeting. During brainstorming, everybody thinks about who should be included and at a team meeting, what we have relatively often, which we always have regularly, all patients that could be included are put forward. | 2.8 |
| **51** | For the normal NIS studies, the exclusion and inclusion criteria are so minimal that I don't have to do much research. | 2.23 |
| **52** | [There are already data in the CIS.] Exactly, exactly, but I still always have to look it up in a doctor's letter. | 2.10 |
| **53** | In principle, it all depends on me, both the selection, the thinking about which patient exists, which patient might be suitable, contacting patients, including patients yes or no, and continuing looking after the patients, all my job. | 2.15 |
| **Interview 2:** | | |
| **54** | Of course, this is very tedious, so the studies usually do not only require one criterion, there are always inclusion and exclusion criteria, i.e. patients must meet multiple inclusion criteria and must not meet multiple exclusion criteria, i.e. I scan them for these criteria, I take the main criterion. | 2.7 |
| **55** | We have a wide variety of examinations, and then every single examination really matters, what kind of people do I actually need? The search for patients is correspondingly time-consuming. | 2.7 |
| **56** | We also have a lot of studies where the patients are currently coming for an examination, then get a certain device, and then e.g. are included into a register. | 2.2, 2.9 |
| **57** | Of course, this is a comparatively easy search for patients, because the patients just come in, we know: I see! So the doctors who make interventions, know that we are looking for different patient groups, so to speak, and then they let us know. | 2.9 |
| **58** | Or we, if we have the time, we often do it so that we always look through the findings of the day and see what has been done, then we go through, is there any patient qualified for any study, for any registry, and then we address them or let someone address them. | 2.3 |
| **59** | We then call him and say: "Today a patient came, he would be qualified for our study." We check beforehand if he meets the inclusion and exclusion criteria, and then the [doctor] approaches him and educates the patient and just asks him if he would like to participate. | 2.3 |
| **60** | Usually we do it for *ourselves*, that we make a documentation so that we also know who we have already identified, who is suitable, who is not suitable, so that is what we keep for ourselves, such a documentation. | 2.17 |
| **61** | That is an additional effort, exactly, which is usually not paid for. […] So, it is usually the case that these studies run alongside the normal work of the doctors. They are all working to capacity anyway. | 2.7 |
| **62** | So, we have ... most patients, you have to say, come to us again and again. That is ... so they mostly have diseases that lead them to the clinic again and again. They are already known [...] But usually we have the patients ... we do not recruit patients who have never been to us before, if you mean so. So, of course we know them. | 2.9 |
| **63** | Then we just told the outpatient practitioners, we said: "We have a study there." We usually give them a paper that says what the study is about, and then they can specifically address patients who are coming and who would be suitable. So, we tell them: "We are looking for the patients." [...] Then they in turn let us know, they say: "We have a patient there." And then we can inform the principal investigator. | 2.3, 2.5 |
| **64** | [Are there regular appointments?] No, unfortunately not. | 2.24 |
| **65** | It's just that the doctors here all have an insane workload and of course we sit down with the individual study doctors. | 2.28 |
| **66** | Typical problems are, of course, that we certainly miss a lot of patients in this way. | 2.11 |
| **67** | I already know what the individual studies are about. So, of course, I know the most obvious inclusion and exclusion criteria. [...] if it really comes down to such details, I can’t manage to have it all in my head. | 2.2, 2.25 |
| **Interview 3:** | | |
| **68** | So, we have a working group in-house that actually selects patients out of everyday clinical practice. | 2.8 |
| **69** | But they are usually not picked out of the [CIS], but then the physicians on the ward basically get a handout and then actually have to look after patients with a given diagnosis and then the patient is asked if he would like to participate in the study. | 2.5, 2.19 |
| **70** | [Is the type of procedures very diversified or can it be said that there is mainly one procedure?] It really depends on the studies. | 2.26 |
| **71** | One working group […] that advertises in local newspapers. | 2.12 |
| **72** | [How high do you estimate is the proportion of what is done from the knowledge of the doctors or the treating staff, and how high the proportion that data research is more likely?] Fifty-fifty maybe. | 2.1, 2.27 |
| **73** | One knows the patients, but of course one still has to look for the Excel list and contact details. | 2.28 |
| **74** | So there really are studies with very restrictive inclusion criteria, where some laboratory values have to be very controlled. And then it's really a matter of luck. | 2.29 |
| **75** | [Can you briefly say where the patients for your studies come from?] (a) Cooperation with resident [specialists], (b) from the patient population here in the clinic, that is, patients with a certain clinical picture, (c) from existing file systems. | 2.2, 2.30 |
| **76** | [Regular meetings?] Nope. | 2.24 |
| **77** | [Personal motivation] Extremely important [...] it takes a lot of heart and soul. | 2.4 |
| **Interview 4:** | | |
| **78** | In the [regular meeting], as a study-center physician, I am, so to say, required to attend there. That is, it is best if I know all the studies that are currently running in our ward. | 2.8 |
| **79** | I have to know the studies so well that I can make study proposals at the [regular meeting]. These study proposals will be discussed [...]. And then it is decided: Yes, could be suitable for the study, or could not be suitable for the study. | 2.1 |
| **80** | This [identifier] number from this suggestion, which I made, is then compared with this calendar management software and is printed out in the form of a report for the study nurse. […] The study nurse can then write in these patients for the following week or whenever the patients have an appointment: "Please think about the study!" | 2.5, 2.31 |
| **81** | And in retrospect I can still bring in patients again via this system, what is going on back there, the patients were not presented in the tumor conference, but again via the scheduling program for a study proposal. | 2.31 |
| **82** | […] we have a study book here, i.e. the general practitioner is involved in himself. This is a study book, where the current studies from each year are always included. It is reissued once a year. There are inclusion and exclusion criteria. | 2.32 |
| **83** | [...] I kept the study tree so up-to-date that I can get information today, i.e. Study is closed, study is not closed, or the study was initiated yesterday or is initiated today, i.e. I promptly change this study tree and put it in a central folder where all doctors and also study nurse and nurses have access, and is linked via the desktop. | 2.32 |
| **84** | [...] keep the study in mind with this study tree and can then go down my tree and can then say: "OK, patient X fits study Y," and then look at the relevant protocol. | 2.1, 2.32 |
| **85** | The doctors are actually very clearly encouraged because they know their patients best and should have a rough overview of the studies. | 2.3 |
| **86** | [...] Setting the course there [...] and priming this patient collective a little, it's not too bad to know the studies and to know this study tree, to use the study book. | 2.32 |
| **87** | I just print out the whole [regular meeting] up here on a sheet of paper and go through all the patients. Most of the time I select over 60 patients. | 2.32, 2.33 |
| **88** | I think that about 70-80% of my study proposals in the [regular meeting] ultimately fit the patient. | 2.6 |
| **89** | In the [CIS] mini medical history, the ward doctors enter a kind of form, where they enter the patient characteristics, the previous therapies and the pathologists in turn enter the histology of the preparation for these patients, and this is then linked. | 2.10 |
| **90** | These have to be entered. The least information is already available in the [CIS]. There is always someone who enters the station. [Regular meetings] are prepared by ward doctors 1-2 days before the [regular meeting]. | 2.10 |
| **91** | [Personal motivation] For the largest, for the absolutely largest factor […]. I can take any patient to where I want to take him, most of them, let's say 90% every patient […] that their own motivation to involve patients in studies depends enormously on the recruitment number. | 2.4 |
| **92** | It really depends on your personal motivation. | 2.4 |
| **93** | And earlier it worked, and recruiting many years ago was […] significantly better without all the systems. | 2.34, 4.20 |
| **94** | So, people know that this is so complex, and that's why you are a little bit deterred by studies from the start. That's a point, […] I haven't found a way to get the doctors to do it anyway | 2.7 |
| **Interview 5:** | | |
| **95** | […] deals with the case file, so he goes to the ward and flips through the case file and looks to see if he finds the criteria […] electronic system, he also goes into the electronic file and looks. | 2.16 |
| **96** | […] quite simply the personal conversation on the ward. | 2.2 |
| **97** | […] Consultation the nursing staff or the doctors. […] But that takes a lot of personnel. | 2.2, 2.3 |
| **98** | Of course, so I don't think it's very modern to do it that way, but that's the most reliable way for us. | 2.6 |
| **99** | What doesn't work is internal cooperation, asking in our ward. | 2.11 |
| **100** | Here in our ward, you have to take care of yourself every day, you get no patients reported [...] you have to look at the criteria yourself every day. | 2.7 |
| **101** | That those in the clinic who have a caring and curing focus often simply do not have this on the radar. They don't think about it. | 2.7 |
| **102** | When we include patients, they are often the previous. | 2.35 |
| **103** | Every scientist has one or more projects and is solely responsible for them and goes through the whole process. | 2.36 |
| **104** | I think that patient identification works well for us. I'm not even dissatisfied with it. I believe that this is also a cumbersome way and very time-consuming, but ultimately the result fits the outcome. | 2.35 |
| **Part 3: IT support – actual state** | | |
| **Interview 1:** | | |
| **105** | On the one hand, we have a [specific diagnosis] register, there the patients are recorded relatively comprehensively, with blood samples and everything. And then you can also research it. We all have it in there, there's an internal research group that looks after it. | 3.4 |
| **106** | [Is this database specific to your department? Especially for clinical studies?] Extra, exactly, especially for studies. | 3.4 |
| **107** | [Do you know if this is also available in other departments?] So, for [own department] I know it, the [other departments] have something where they are still building up. But that's pretty specific because we have a [specific diagnosis] focus. It's not in the lab data. You have to look up that in surgical reports, and most things are information that results more from the patient's medical history; has he ever been infected with this or that virus, yes or no? All of this would have to be entered into a database, so to speak. | 3.4 |
| **108** | If you take the whole inclusion and exclusion criteria from all the studies together, you could theoretically document everything that could be documented, and then you would have to include every patient in this regard with all this data, that maybe he could come into a study at some point. | 3.4, 3.13 |
| **109** | But for complex patients, where it becomes rather extensive, you would have to enter that into a database or separately again for everyone. | 3.11 |
| **110** | And these are recurring inclusion / exclusion criteria in many studies, which are very similar, of which it could be operationalized, but would be an insane effort. | 3.5 |
| **111** | [Free text doctor's letters] Cannot be evaluated at all. | 3.2 |
| **113** | Except that you do your own research in the CIS system, no. | 3.1 |
| **114** | I don't have a database where I can search for criteria. | 3.12 |
| **115** | I have of course already selected according to diagnoses in [CIS]. However, these diagnoses are not necessarily the diagnoses that I now have in a doctor's letter. That is the problem. | 3.13 |
| **Interview 2:** | | |
| **116** | With us all findings from the examinations are stored in a directory. In principle, these are Word documents, […] They will of course be digitized at some point and then be in [CIS]. | 3.2, 3.15 |
| **117** | In the end it is always the case that we have to read the doctor’s letter again. | 3.14 |
| **118** | [Is the data that you already have in the HIS, are they suitable to support you in their current form?] Yes, of course, it is. | 3.1, 3.8 |
| **119** | The good thing is ... what we are always interested in ... for example that the whole blood values can be seen at a glance. This is important for us if we go through such inclusion and exclusion criteria. Then I open the [CIS], look into it, maybe see at first glance: in which other clinics are the patients regularly available as well. […] If we have time, we browse through the reported findings and see if any patients are suitable for certain registries. | 3.1 |
| **Interview 3:** | | |
| **120** | [Little search in inventory data, medical reports or CIS?] No, not in clinical information systems. | 3.9 |
| **121** | Then there is a small database for each clinical picture; partially it's just some Excel lists or something. | 3.3 |
| **122** | So, we're not searching in [CIS]. So, I don't think that has ever been done in our house. I mean, it is of course conceivable that you - I don't know exactly how far you can go in [CIS] searches, that you say: "Vomit everybody with diagnosis XY." - I've never tried that myself, and I think in our ward... nobody does that. | 3.9 |
| **123** | Every working group has a list of patients who have already participated in something. And, of course, you can search there. | 3.7 |
| **124** | [They created a specific solution themselves?] Exactly. | 3.15 |
| **125** | Everything has grown historically, and these working groups have been on the road for many years and they almost always work with their own databases. | 3.15 |
| **126** | So, we have already worked with [EDC system] […] that you simply - basically, that's great - just ask: "Vomit everyone who has been seen here within so many kilometers and who has and this diagnosis. " And then you get a wonderful list that you can get out as a CSV. | 3.3 |
| **127** | We only used it relatively rarely, and there are political reasons for that. […] Because, we have assured the patient that we will not pass on their data, so that we cannot simply give it to anyone […]. So, we explicitly promised the patient that we would not do this. | 3.16 |
| **128** | Of course, this is a certain effort, as I said, politically not unproblematic. | 3.5 |
| **Interview 4:** | | |
| **129** | [Feasibility] We use data, databases, which, for example, if we are now studying [subject], we use the [special database]. | 3.3, 3.4 |
| **130** | [Recruiting] We register this in a database, this study proposal, and the following advantage that we enter the common identifier that we use here, namely [identifier], and we get it once a week ... we get an export from the scheduling program of all patients. We have approximately 700 outpatient patients from Monday to Friday. | 3.17 |
| **131** | I can then also call up this documentation sheet in [CIS], i.e. which is saved and printed there and signed by my boss as real source data. | 3.1 |
| **132** | The least data is available, so you can if I have already entered a [regular meeting]. | 3.6 |
| **133** | [... in the CIS] certain information is taken over repetitively from the last entry so that I do not have to re-enter it. | 3.18 |
| **134** | We produce recruitment numbers from our studies once a month, where we have our clinical database where all study patients are registered. | 3.4, 3.15, 3.19 |
| **Interview 5:** | | |
| **135** | [Feasibility] Actually, I use my own internal data to calculate it | 3.20 |
| **136** | [Recruitment] In the electronic system, he also goes into the electronic file and looks up. | 3.2 |
| **137** | [CIS] Because we do not know it. | 3.10 |
| **138** | We […] have difficulty accessing this electronic data. | 3.21 |
| **139** | Often we don't get any rights to see this, so even if we ask that we only have read rights - we don't want to document anything in the patient record, that's totally okay, but we would like to be able to read it. | 3.21 |
| **140** | [...] we don't even know certain systems, electronic documentation systems at all - so I only know the [CIS] - this is actually not really available as a resource. There is a huge barrier for us scientists here in the house. | 3.10 |
| **141** | We still use data from [Lab IS] […] because we have to look up microbiological results | 3.13 |
| **142** | We don't have electronic doctor’s letters at our ward yet, we don't use them. | 3.23 |
| **143** | But we also have [...] a set of basic data integrated in the electronic documentation, which we then export and then continue to work with this database. | 3.22 |
| **144** | But, only because of the good cooperation and preparatory work, we only have the opportunity to enter the data electronically in the first place without double documentation. […] That was a milestone for us for research | 3.24 |
| **145** | It was a bit difficult at the beginning to find defined criteria and cells, from which we then query this data, this information, but in the end it worked well. And I would do that again. | 3.25, 3.26 |
| **Part 4: IT support – request** | | |
| **Interview 1:** | | |
| **146** | Not easy ... Not easy. It would be ... but already possible. It would be possible, e.g. in the HIS system or if you were to write it as an interface, it would be helpful... on the one hand, to differentiate between these study patients who are included into a NIS, which are therefore easy to recruit, or a study who practically does almost everything and we have relatively little effort, and the patient, where we have a lot of effort. That we would encrypt patients in the system with quasi binary code with regard to individual aspects, be it now diagnosis, which of course is always a decision criterion in the whole studies, the patient must and must not have the diagnosis… | 4.4, 4.5 |
| **147** | [A lot of data, what you just said, diagnoses, is already available. If there was anything, would it be helpful?] Yes. Definitely. Definitely, because we have so many patients. And if there was such a thing or if I could wish for something: we take over certain criteria that are stored operationalized. | 4.5 |
| **148** | From study to study, they do not differ that much, and the studies are often structured similarly, so that it could be filled relatively easily with individual fields or database fields and sorting the patients according to it, that would be incredibly helpful. | 4.5 |
| **149** | That I would now have a ... that in the [CIS] like in the doctor's letter that is quasi evaluated or marked with fields "main diagnosis", so that I can assign it immediately using the ICD code. | 4.1 |
| **150** | Then with one click I could ... I could select 90% of the patients correctly. | 4.1 |
| **151** | […] that on the one hand you have the opportunity to carry out a search yourself, on the other hand such suggestions also come, firstly for our study team, who sometimes make a preselection. | 4.3 |
| **152** | The main diagnosis must always be kept up-to-date, and that would of course be important to keep the doctor's letters up-to-date. | 4.13 |
| **153** | But that is a prerequisite, and then that you have to meet some of the criteria, let's say, the really hard ones, which already decide about 90% of the patients. That you can select them well, maybe 10, 15 in total, which would be a great help. | 4.5 |
| **154** | ... every patient who has a main diagnosis like this, of course, has to appear somewhere in a database field and with a YES / NO query or whatever, that you can select that. But there are possibilities that would help us extremely. | 4.1, 4.5 |
| **155** | If only one had a speech recognition system […]. | 4.17 |
| **156** | So, there would be many options. A clever mind would have to sit behind it and go through it individually with the colleagues from the [department] and then quasi operationalize it. And then a database. Then you could do a lightning search. | 4.1 |
| **157** | So, it only benefits everyone. Win-win situation, there is nothing that would be a disadvantage. | 4.5 |
| **Interview 2:** | | |
| **158** | That e.g. something that I would also be interested in if you can incorporate a feature that makes it much easier. But that's what it's about? So I think it's a very interesting project. | 4.4, 4.6 |
| **159** | It would be easier to type in a keyword somewhere and then it would vomit a result. The problem is, of course, that the criteria are so different that I don't think anybody can develop anything where we are prepared for all cases. | 4.1, 4.9 |
| **160** | In the end it is always the case that we now have to read the doctor’s letter again. See what kind of disease the patient has… I think it is impossible to grasp all of this. | 4.4 |
| **161** | [Anything that can be taken from you would be helpful?] That would be great. So that would be a time saver. | 4.5, 4.11 |
| **162** | The question is always: can this be implemented at all for data protection reasons? | 4.7 |
| **163** | If of course you had a huge file now if I had a huge file where I could enter certain things and it would at the end vomit the patients who met all of these criteria, that would of course be fantastic. | 4.1, 4.5 |
| **164** | But that would mean that now not only doctor’s letters have to be deposited in the [CIS], but also certain things still have to be deposited, additionally. | 4.8 |
| **165** | If someone would do that for us, this search, and e.g. an email would always come early: "Yesterday there were 5 patients who received this and this device." | 4.2 |
| **166** | Sure, if you could automate that, it would be great. | 4.5 |
| **167** | Of course, I now have very different studies and registers in my mind, so that should be answered really differently, because for some studies the inclusion periods are also very, very short, just. It would bother me now if you gave me half a year ... well, one would simply have to say: "So, it's over, recruiting is over," and stop the whole thing again. Yes, I can also imagine that. Can I ... especially for such registers I can imagine that very well. | 4.9 |
| **168** | But if we now want to fall back on old patients for new studies, they may of course not have ... this criterion that we need now. That there is always a certain limitation of course there is always. | 4.18 |
| **Interview 3:** | | |
| **169** | I think that the things that you really need cannot be implemented at all, so they really cannot be implemented. | 4.4 |
| **170** | So especially if you work together with private practices, we cannot access their data, because their computers with patient data are not connected to the network for data protection reasons. So you can't look at it from here, and even if you could, it would be difficult to go there and say: "Dear doctor XY, we saw with 'spyware' that you have this or that patient there, we would like to get them." So that's completely utopian.. | 4.7, 4.12 |
| **171** | For some things, I don't find it wrong to search in the hospital information system. | 4.1 |
| **172** | [In your opinion, what would be a more suitable or better system, or does it make no difference?] I think a query system because then you ask when you really need people. So if I don't have a study at all or have already recruited enough or something, then I don't want to be spammed with any emails that John Doe is lying around with an XY diagnosis. | 4.3 |
| **173** | As long as this study is ongoing and as soon as it is completed or fully recruited, you quasi close it and then you will not be contacted. Yes, of course that also sounds like a clever plan. True, is actually not a wrong idea. | 4.2 |
| **174** | Of course, that depends on the number of cases. If the emails pop up in 5-minute intervals, then it makes no sense anymore, then a bulk email. But the more common case is probably that this is rather rare, and then you might really want to know immediately. […] Extremely study-dependent, yes, and depending on the inclusion criteria, as required. | 4.10 |
| **Interview 4:** | | |
| **175** | I don't know of any tool that would make work easier, right? | 4.19 |
| **176** | If you already have data from the start that I still have in printed form, i.e. still on paper, when I generally enter such data electronically, then of course I can put an algorithm behind each study, which then contains certain variables queries, combined with each other and automatically makes suggestions. | 4.1, 4.8 |
| **177** | The study proposal comes automatically without anyone having to think about a study if this entry has already been made during the patient admission process. | 4.8 |
| **178** | Then flashes, for example study proposal X or Y. | 4.2 |
| **179** | Very few things will run so automatically that you make a request and that the system is completely there in its perfect perfection, i.e. it takes a lot of time and money. | 4.21 |
| **180** | Whether I really need a tool for this - I don't believe it. Honestly, I don't think so. | 4.20 |
| **Interview 5:** | | |
| **181** | Where we would often like more support with regard to IT, that it can perhaps be queried much more consistently, which would of course make it easier if we could get to know these systems and would know, these things are also documented. | 4.1 |
| **182** | […] we can do it ricky-tick from our workplace, or maybe even set up an electronic reporting system that we always get a “pling” on the screen when a patient meets certain criteria. | 4.2 |
| **183** | It would of course be really convenient if I didn't have to search through this electronic documentation myself anymore, but if there was a programmed tool that simply queries certain fields and data at defined times or in defined periods, and whenever there is just a certain result comes out, I get a "pling" on the screen, […] That would be extremely great. | 4.2 |
| **184** | And then, perhaps according to our most common criteria, one would have to adapt the electronic documentation. | 4.8 |
| **185** | Then the documentation has to come first so that it can be requested and we just get a message. That would be great. | 4.5, 4.8 |
| **186** | [Active solution] Yes. | 4.2 |
| **187** | [Self-paced] […] That could also be. So just to determine the number of cases, but if it were […] there would be a significant added value if we could ensure that there were cross-departmental collaborations. | 4.3 |
| **188** | So, if I got a message in the morning, 5 patients can be generated in the clinics in question, who at least meet the hard criteria, I can still find out the rest. | 4.3 |
| **189** | But if that were continuously and I was notified at all times, that would be a major advantage. | 4.2 |
| **190** | That would be very profitable, precisely because we expect fast progress and rapid changes in the general condition. | 4.2, 4.5 |
| **191** | [Alert Fatigue] I believe that this is totally unlikely with us because the station capacity is small, [...] that I would accept that. | 4.16 |
| **192** | Because I think the problem of these 10-minute memories or something becomes relevant if you recruited at many stations, […] It might just be nicer if you bundled it up and said you get 2 in the morning and at noon sometimes a report bundled with everyone, but for [...] critical patients it would be totally okay for me if I would always report because I knew that would never flood me. | 4.15, 4.16 |
| **193** | I would like to advocate that even if there are very different databases here in-house or in positions and institutions that maintain different data, that you can actually merge them, that you strengthen these interfaces again. | 4.14 |
